# Supplementary material for: Arabidopsis Ubiquitin-Conjugating Enzymes UBC7, UBC13, and UBC14 Are Required in Plant Responses to Multiple Stress Conditions
Source: Plants (Basel). 2020 Jun 8;9(6):723. doi: 10.3390/plants9060723 (PMC7355981; doi:10.3390/plants9060723)
Supplement: Supplementary file 1 [file plants-09-00723-s001.pdf]

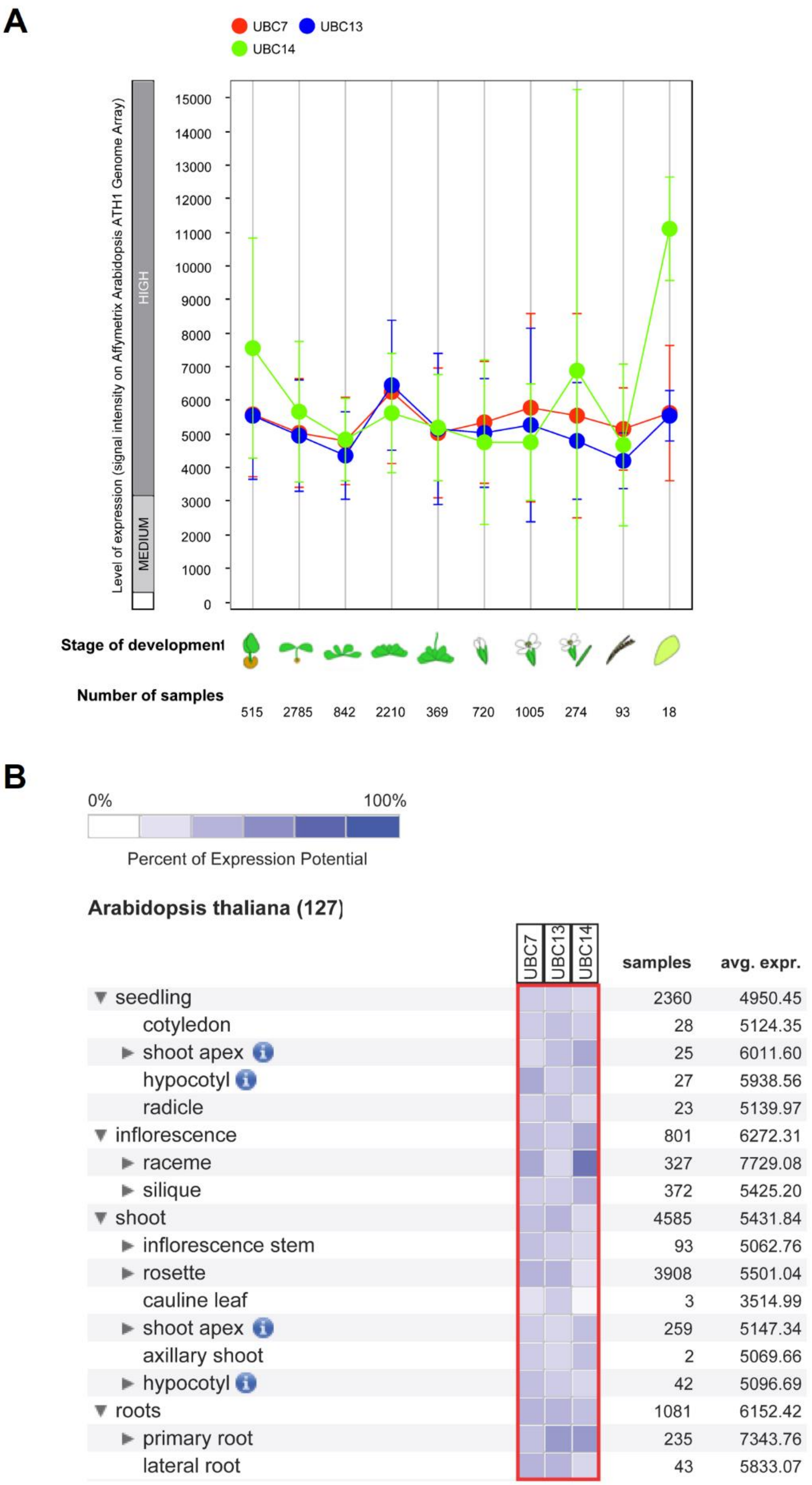

Figure S1. Expression profiles of *UBC7*, *UBC13* and *UBC14* at different developmental stages (A) and in different tissues (B). The figures were generated by Genevestigator based on microarray expression data (https://genevestigator.com).

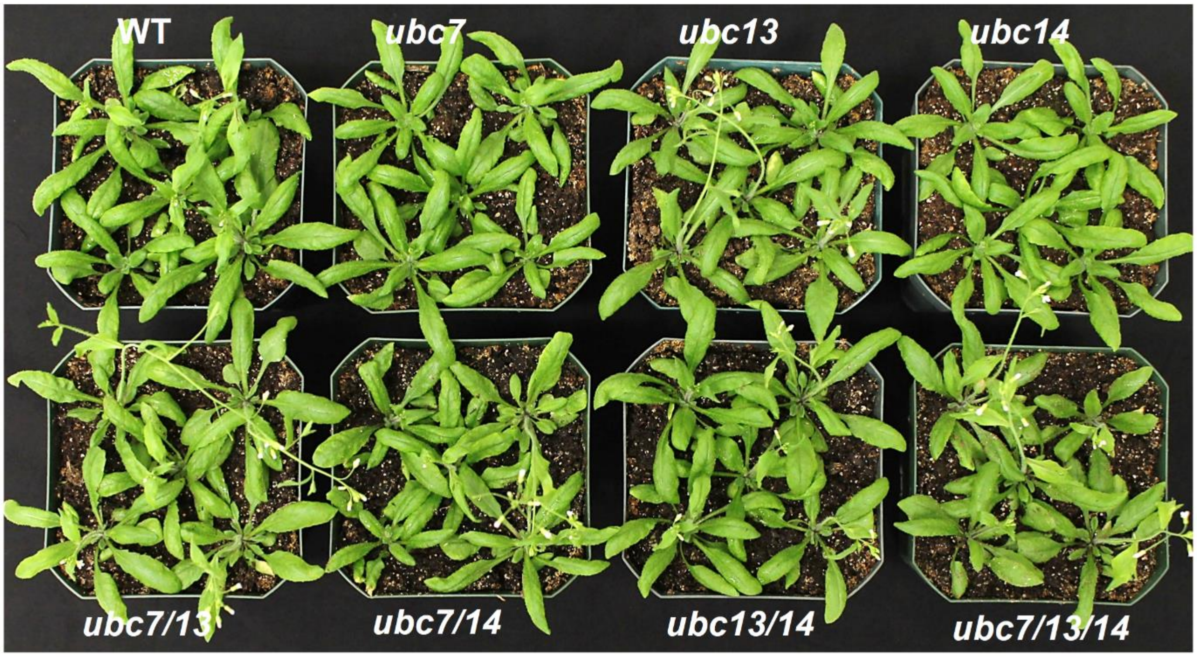

Figure S2. WT and mutant plants grown in soil under normal conditions. WT, *ubc7*, *ubc13*, *ubc14*, *ubc7/13*, *ubc7/14*, *ubc13/14* and *ubc7/13/14* mutant seeds were planted in pots. The image was taken on the 30th day after seed planting.

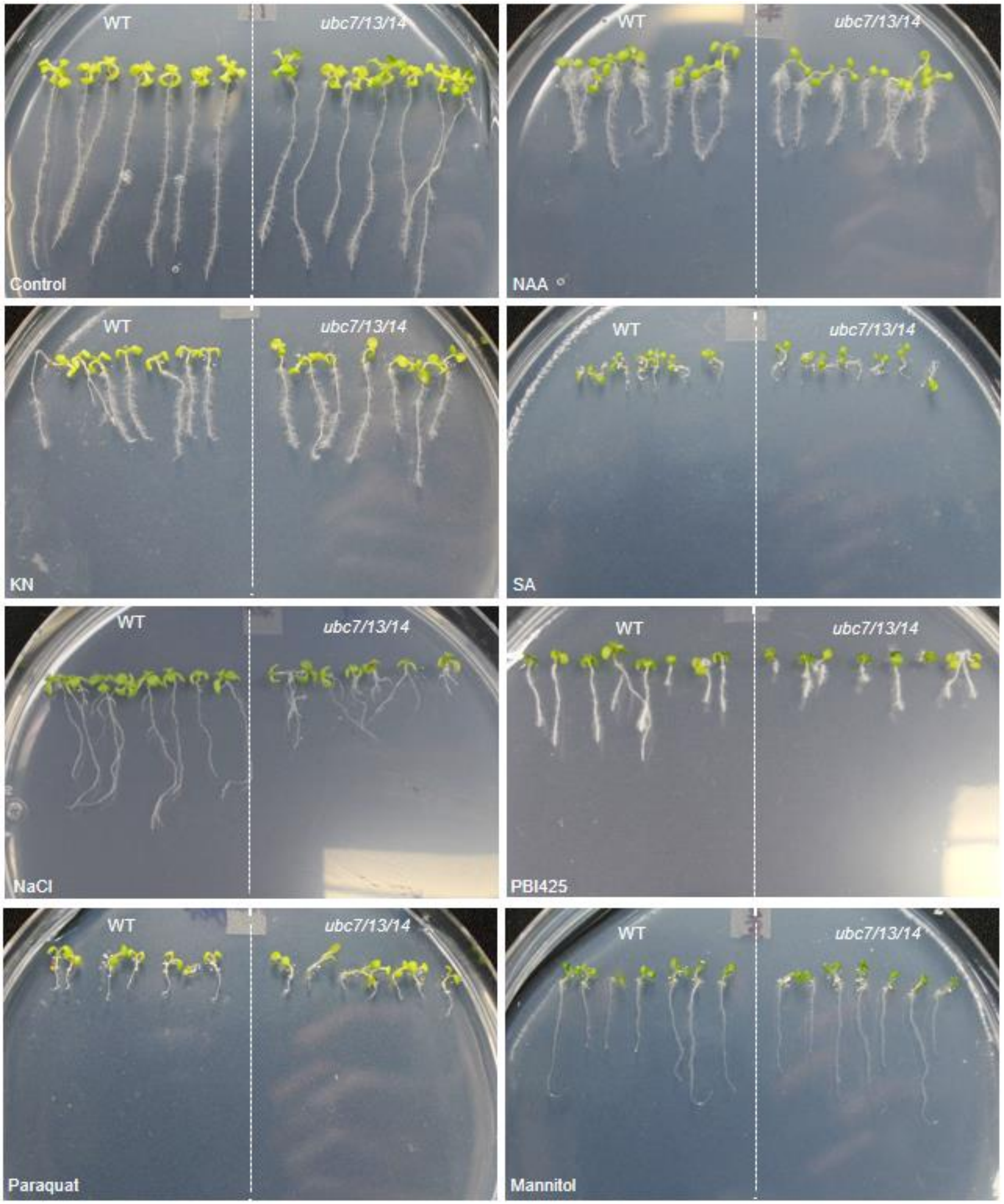

Figure S3. Initial phenotyping screening. The WT and *ubc7/13/14* mutant seeds were sterilized, stored at 4° C, and then plated onto the normal (1/2 MS + 1% sucrose + 0.7% agar) or treatment plates supplemented with the following: 0.05 μM naphthaleneacetic acid (NAA), 2.5 μM kinetin (KN), 100 μM salicylic acid (SA), 120 mM NaCl, 0.10 μM PBI425 (an abscisic acid analog), 0.05 μM paraquat and 4% mannitol. In each plate, seven WT and seven mutant seeds were plated side by side. Images were taken on the 12th day after seed plating.

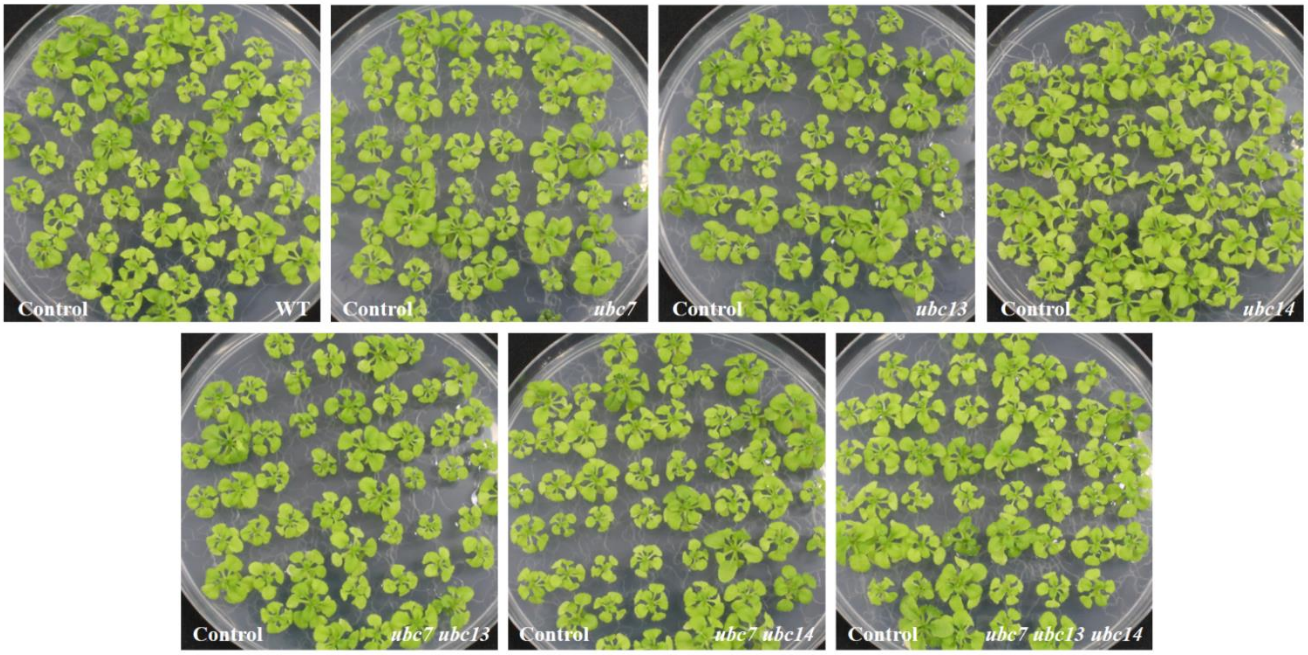

Figure S4. Growth of single to triple mutants of *UBC7, UBC13* and *UBC14* on normal plates. Arabidopsis WT and mutant seeds were sterilized, stored at 4° C, and then plated on ½ MS plates. The plates were placed in a tissue culture chamber and images were taken after 21 days.

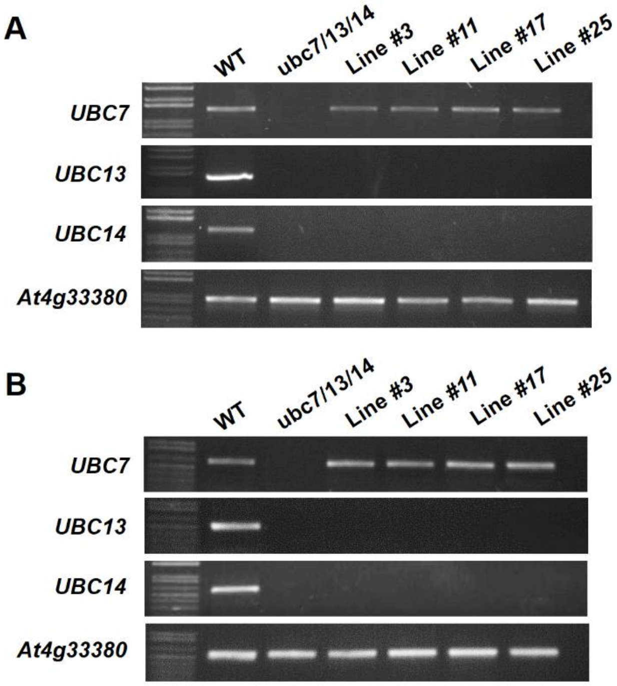

Figure S5. Genotyping of WT, *ubc7/13/14* mutant and complementation lines with a genomic *UBC7* fragment. A *UBC7* genomic fragment (containing the region of 1957 base pairs before ATG, the coding region and 667 base pairs after the STOP codon) was introduced into the triple mutant. Four independent complementation lines (indicated by the line numbers) were analyzed.
(**A**) Genomic PCR for genotyping. Gene-specific primers (see Table S1) were used to detect the presence of genomic DNA of *UBC7*, *UBC13* or *UBC14*. A *UBC7* band is present in the complementation lines, but absent in the triple mutant.
(**B**) RT-PCR analysis of WT, *ubc7/13/14* mutant and four complementation lines. Gene-specific primers were used to detect of the presence of *UBC7*, *UBC13* or *UBC14* transcripts. The gene *At4g33380* was used as a reference and shown at the bottom. The plant lines analyzed are indicated above the panels.

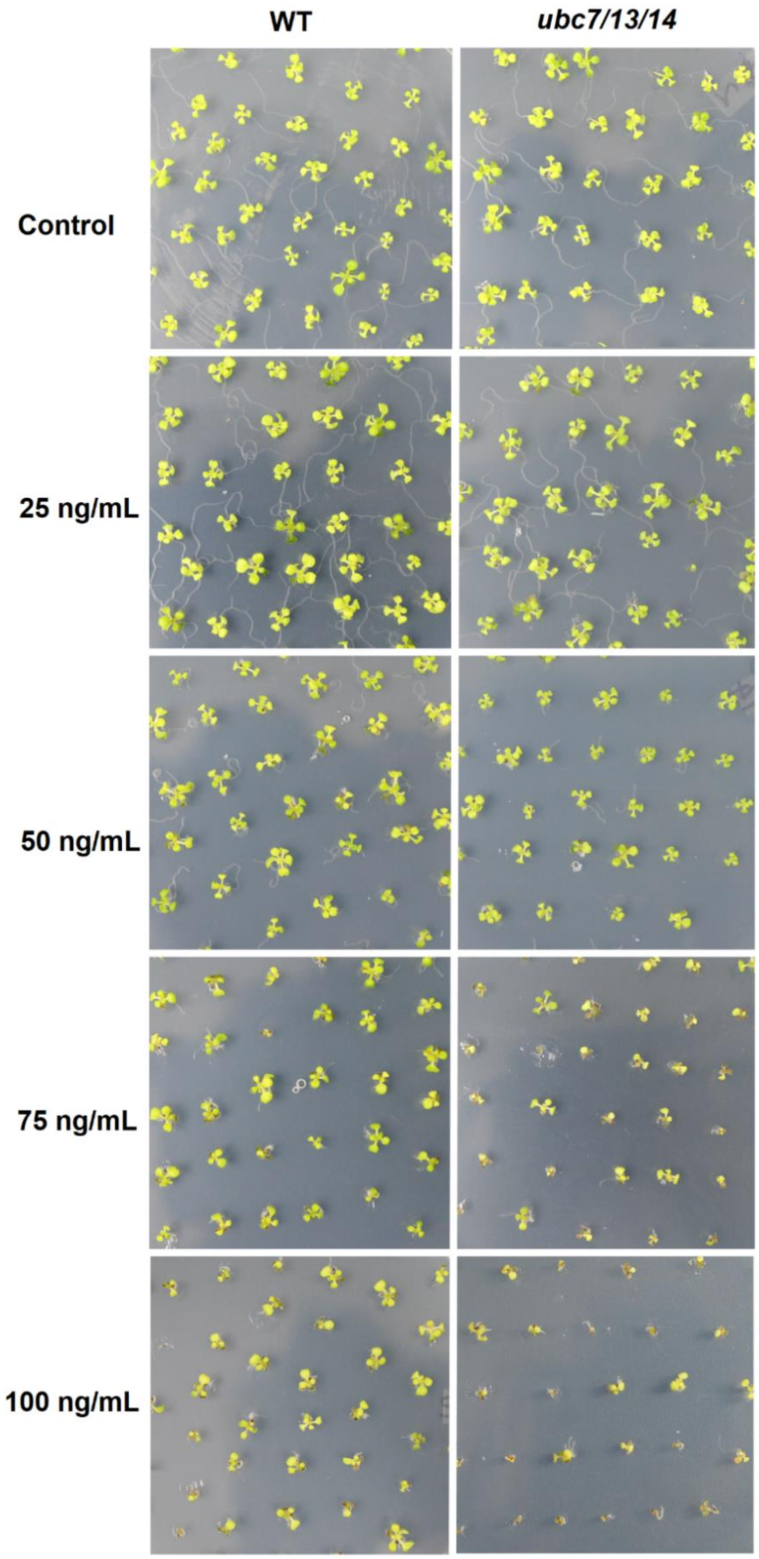

Figure S6. Sensitivity of the *ubc7/13/14* seedlings to tunicamycin. The WT and *ubc7/13/14* mutant seeds were sterilized, stored at 4° C, and then plated onto the normal or treatment plates with different concentrations of tunicamycin (each plate having 50 seeds). The plates were placed in a tissue culture chamber. Images were taken on the 12th day after seed plating. Tunicamycin concentrations used are indicated at the left side of the panel.

Table S1. List of primers for mutant genotyping and RT-PCR

Primer ID	Purpose	Primer sequences
HW1317	*UBC7*, forward	CAGTGTCGACAATGAAAACATCGATTCCCGA
HW1278	*UBC13*, forward	CAGTGTCGACAATGAACTCACAAGCTTGTC
HW744	*UBC7* and *UBC13*, reverse	TCAAAACATTTCTTGAGACTTTC
HW1279	*UBC14*, forward	CAGTGTCGACAATGGCTAATAACCAAGCAAG
HW1280	*UBC14*, reverse	CAGTGCGGCCGCTCATAGCATCTCTTGCGAT
HW499	T-DNA primer for *ubc7-1, ubc14-1*	CCGCAATGTGTATTAAGTTGTC
HW1015	T-DNA primer for *ubc13-1*	AACGCTGCGGACATCTAC
HW471	*AT4G33380*, forward	ATGAGAAGCTGGAGGAAGC
HW472	*AT4G33380*, reverse	TCAAGCCGTTACAACACC
